# Sex differences in arterial stiffness in a rat model of type 1 diabetes

**DOI:** 10.3389/fphys.2026.1802411

**Published:** 2026-03-20

**Authors:** Swasti Rastogi, Amanda A. de Oliviera, Yingnan Zhai, Jessica Liaw, Linxia Gu, Kenia P. Nunes

**Affiliations:** 1 Laboratory of Vascular Biology, Department of Biomedical Engineering and Science, Florida Institute of Technology, Melbourne, FL, United States; 2 Laboratory of Biomechanics, Department of Biomedical Engineering and Science, Florida Institute of Technology, Melbourne, FL, United States

**Keywords:** arterial stiffness, atomic force microscopy, diabetes, pulse wave velocity, sex differences, extracellular matrix

## Abstract

**Background:**

Individuals with type 1 diabetes (T1D) exhibit elevated arterial stiffness and are at a higher risk of developing cardiovascular disease, both of which are sex dependent. However, whether sex differentially affects aortic structure at macro-, micro-, and nano-levels, which represent evaluations of the aorta at multiple scales, remains poorly understood. Therefore, we investigated sex-based differences in arterial stiffness by assessing pulse wave velocity (PWV), extracellular matrix remodeling, and atomic force microscopy (AFM) based medial-layer biomechanics in the aorta of a rat model of T1D.

**Methods:**

Male and female Sprague Dawley rats were injected intraperitoneally with streptozotocin (65 mg/kg) to induce T1D. After 4 weeks, arterial stiffness and vascular alterations were evaluated in aortas of both sexes across multiple levels. At the macro-level, *in vivo* arterial stiffness was measured using ultrasound-based PWV. At the micro-level, structural remodeling was evaluated by quantifying collagen and elastin content in the aortic extracellular matrix. At the nano-level, biomechanical properties were assessed using AFM to determine Young’s modulus in the aortic tissue.

**Results:**

T1D increased *in vivo* PWV in both sexes, with significantly higher PWV in diabetic males compared to diabetic females. Structural analysis revealed that diabetic males exhibited higher collagen deposition than diabetic females, whereas the Young’s modulus increased with diabetes but showed no differences associated with sex.

**Discussion:**

These findings suggest that sex differences in T1D-related arterial stiffness at the macro level are primarily associated with extracellular matrix remodeling rather than nanoscale vascular smooth muscle cell stiffness at this disease stage.

## 1 Introduction

Diabetes, a chronic condition caused by the body’s inability to produce and/or properly use insulin, affects more than 800 million people worldwide (World Health Organization, 2024). In 2021, diabetes was the direct cause of more than 1.6 million deaths, with approximately 850,000 occurring before the age of 70 (World Health Organization, 2024). While type 2 diabetes (T2D) is the most prevalent, T1D remains a significant challenge, as neither its cause nor a means of prevention is currently known (World Health Organization, 2024). Patients with diabetes, regardless of type, are at increased risk of developing cardiovascular disease (CVD) (Rosengren and Dikaiou, 2023). Notably, in patients with T1D, traditional CVD risk prediction models perform poorly in anticipating CVD events, suggesting the presence of disease-specific underlying factors (Zgibor et al., 2006). Arterial stiffness, a subclinical marker of CVD risk, can predict mortality in patients with T1D (Tynjala et al., 2020), indicating that it is an important prognostic factor.

Ultrasound-based pulse wave velocity (PWV) has therefore been widely used in clinical studies to assess arterial stiffness in both types of diabetes (Prenner and Chirinos, 2015). In addition, a few studies have employed atomic force microscopy (AFM) to achieve nanoscale resolution of the biomechanical properties of diabetic vascular tissues, providing unique insights into nano-level structural changes associated with the disease (Akhtar et al., 2014; Targosz-Korecka et al., 2017; McCallinhart et al., 2020). The extracellular matrix (ECM), particularly the balance between collagen deposition and elastin degradation, plays a central role in determining arterial stiffness (Lacolley et al., 2017). In parallel, changes in vascular smooth muscle cell (VSMC) mechanics further contribute to vessel rigidity in diabetes (Akhtar et al., 2014). Together, these cellular and extracellular alterations may shape the biomechanical properties of the diabetic vasculature. Despite these advances, a critical gap remains in our understanding of whether biological sex influences vascular biomechanics in diabetes.

Therefore, in this study, we investigated the impact of T1D on aortic stiffness in males and females and examined whether changes in *in vivo* stiffness are associated with the combined effects of ECM remodeling and VSMC stiffening or is predominantly driven by one of them.

## 2 Materials and methods

### 2.1 Animal model and experimental design

Male and female Sprague Dawley rats (total n=24; 6 animals per group) were obtained at 6–7 weeks from Taconic Biosciences. Animals were housed in pairs of the same sex with unrestricted access to water and food, and allowed to acclimate for at least one week before experimentation (Percie du Sert et al., 2020). T1D was induced by a single intraperitoneal injection of streptozotocin (STZ; 65 mg/kg; MilliporeSigma, Burlington, MA, United States). Control animals received a single intraperitoneal injection of vehicle (citrate buffer, pH 4.5). T1D was confirmed by fasting glucose levels ≥250 mg/dL one week after the injection. After 28 days, blood pressure was measured using the non-invasive tail-cuff method (High-Throughput CODA System, Kent Scientific Corporation, Torrington, CT, USA) and aortic PWV was assessed (see below), after which rats were sacrificed by exsanguination via cardiac puncture under inhaled isoflurane anesthesia (4% in 100% O_2_). Then, aortas were dissected, immersed in cold physiological salt solution, cleaned of surrounding fat tissue, and snap-frozen embedded in Tissue-Tek^®^ Optimal Cutting Temperature (OCT) Compound (Sakura Finetek Inc., Torrance, CA, USA) for cryosectioning.

### 2.2 *In vivo* assessment of arterial stiffness in aortas using pulse wave velocity

PWV was measured using a Vevo3100 ultrasound system (FUJIFILM VisualSonics, Toronto, ON, Canada) equipped with an MX250 transducer. In brief, rats were anesthetized with 1–3% isoflurane, maintained at 1.5–2% during imaging, and placed supine on a heated (37 °C) platform with ECG electrodes for monitoring. The abdominal aorta was imaged longitudinally, and PWV was calculated using the transit-time method. The time delay (Δt) between two selected aortic sites was determined from the foot of the Doppler waveform relative to the ECG R-wave. Distance (d) between the sites was measured directly, and PWV was calculated using VevoLab Software (version 5.8.1, FUJIFILM VisualSonics, Toronto, ON, Canada) as:


PWV = d / Δt

Following this, the aorta was imaged longitudinally in long-axis view using B-mode to evaluate the aortic internal diameter; three measurements were taken per animal, and the values were averaged per animal.

### 2.3 Determination of collagen deposition and elastin density in aortas

Collagen deposition was assessed using a Picrosirius Red staining kit (Abcam, Cambridge, MA, USA, ab150681) following the manufacturer’s protocol. Brightfield images were obtained at 10× magnification (Zeiss AxioSkop-2 MOT microscope, Dublin, CA, USA), and collagen content was quantified in ImageJ as the percentage of red-stained area within the media layer and the total vessel. For elastin analysis, thawed sections were washed in 1× PBS to remove OCT and mounted with VectaShield Antifade Medium (Vector Laboratories, Newark, CA, USA, H-1000). Elastin autofluorescence was captured at 488 nm using 4× and 10× magnifications. ImageJ was used to quantify elastin autofluorescence (arbitrary units), and fiber fragmentation was evaluated manually by counting breaks in elastic fibers per section at 10x magnification. Two sections per animal were analyzed, and values were averaged per animal. All elastin images were assigned numerical identifiers and elastin fragmentation was independently analyzed by an investigator across all samples. Diabetes-induced ECM remodeling in diabetic animals was also quantified as the absolute difference (delta, Δ) between diabetic animals and controls (Δ = STZ - mean control value for each sex). This approach was used to normalize for baseline sex-specific differences in ECM and to assess diabetes induced changes relative to controls.

### 2.4 *In vitro* assessment of arterial stiffness in aortas using atomic force microscopy

AFM-based nanoindentation is a technique for measuring the mechanical properties of biological tissues using a nanometer-scale tip to determine Young’s modulus. The nanoindentation of control and T1D aortas was performed as previously described by our group (de Oliveira et al., 2022; Rastogi et al., 2025b). In brief, force-indentation curves were obtained in aortic cryosections (10 μm thickness) using the JPK ForceRobot 300 AFM instrument (Bruker, Billerica, MA, USA) coupled with the Bruker Scanasyst-Fluid probes (tip radius: 20 nm, spring constant: 0.7 N/m, resonance frequency: 150 kHz). The scanning location was determined with the ZEISS Axio Observer inverted microscope (Dublin, CA, USA). During the test, aortic sections were submerged in 1x PBS. Three cryosections were obtained from each animal, and one site was arbitrarily selected within the medial layer of each section for AFM scanning (8x8 matrix, 5x5 μm^2^ scanning area). As the tunica media of the aorta is mainly composed of VSMCs, stiffness measurements obtained from this region were considered reflective of intrinsic VSMC mechanical properties. The Young’s modulus (i.e., stiffness) was obtained by fitting the force-displacement curves to the Hertz Model using the JPK PD software (Bruker Nano Inc., Santa Barbara, CA, USA; version 6.1.166). Force curves with an incorrect indentation and retraction pattern were removed from the analysis.

### 2.5 Statistical analysis

GraphPad Prism software version 10.4.1 (GraphPad Software, San Diego, CA, USA) was used to analyze the data, which are shown as means ± SEM. Data normality was evaluated using the Shapiro-Wilk test, and the Grubbs test was used to identify outliers. The group-size difference reflects the exclusion of identified outliers and experimental failures during slide preparation for the ECM and AFM tests. Data were analyzed with a two-way ANOVA with a subsequent Sidak’s *post hoc* test; p<0.05 was considered statistically significant, and *n* represents the number of animals used per group.

## 3 Result

### 3.1 Sex-dependent alterations on macro-level stiffness and vascular diameter in T1D aorta

Body weight was lower, while fasting glucose levels and blood pressure were higher, in diabetic animals compared with their sex-matched controls (**Table 1**).

**Table 1:** Animal profile in control (CTL) and streptozotocin (STZ)-induced diabetic male and female rats.

Outcome	Male		Female	
	CTL (n=6)	STZ (n=6)	CTL (n=6)	STZ (n=6)
Final body weight (g)	230.6 ± 4.8	210.0 ± 7.3*	200.0 ± 5.3	165.0 ± 2.6*
Glucose levels (mg/dL)	98.3 ± 4.2	402.5 ± 22.5*	100.0 ± 4.1	385.0 ± 13.5*
Mean arterial pressure (mmHg)	95.6 ± 1.6	110.8 ± 2.7*	90.6 ± 5.7	105.0 ± 2.7*
Systolic blood pressure (mmHg)	115.5 ± 3.2	136.0± 2.0*	112.6± 4.9	127.9 ± 3.5*
Diastolic blood pressure (mmHg)	85.8± 1.5	98.4 ± 3.1*	79.8 ± 6.3	93.7± 2.5*

Data are shown as means ± SEM. *p<0.05 vs CTL, using a two-way ANOVA with Sidak’s *post-hoc* test.

**Figure 1:**
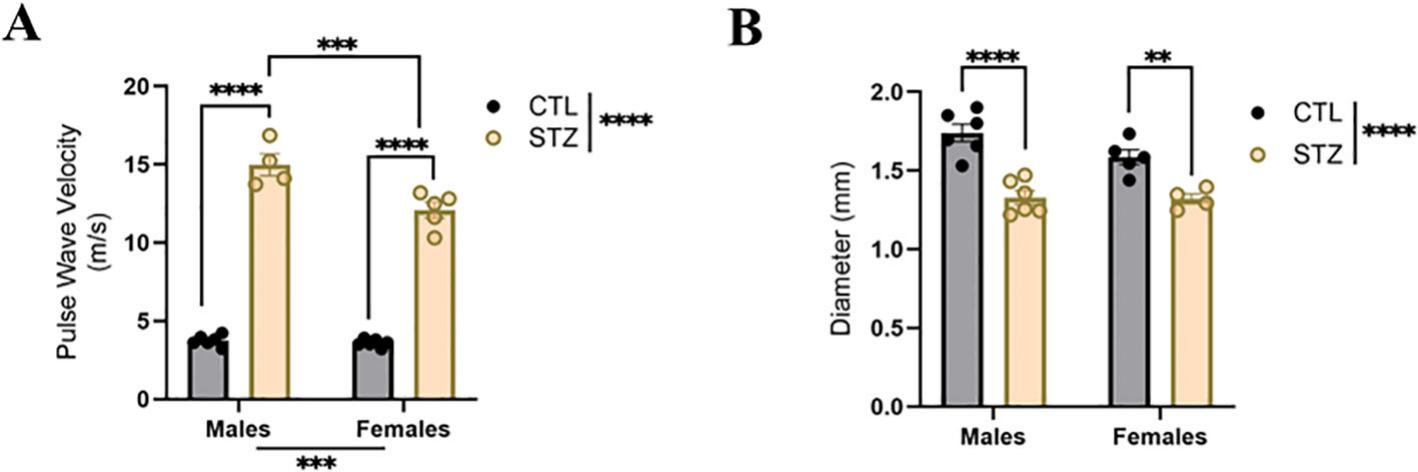
Type 1 diabetes-associated sex differences in aortic arterial stiffness and internal diameter. **(A)** Pulse wave velocity (PWV, as measured by the transit time) and **(B)** internal diameter in aortas of control (CTL) and streptozotocin (STZ)-induced diabetic male and female rats. Data are shown as mean ± SEM; **p<0.01, ***p<0.001, and ****p<0.0001 using two-way ANOVA with Sidak’s *post hoc* test. *n* = 4–6 per group.

*In vivo* PWV, as measured by the transit-time method, was significantly increased in diabetic animals compared with controls (**Figure 1A**). Within the diabetic group, PWV was higher in males than in females, indicating greater systemic arterial stiffness in diabetic males (**Figure 1A**). No sex-based differences in PWV were observed among control animals (**Figure 1A**).

Aortic internal diameter was decreased in diabetic animals compared with controls, with no significant differences between diabetic males and females (**Figure 1B**). In control animals, diameter did not differ between males and females (**Figure 1B**).

### 3.2 Characterization of ECM remodeling in the T1D aorta of males and females

**Figure 2:**
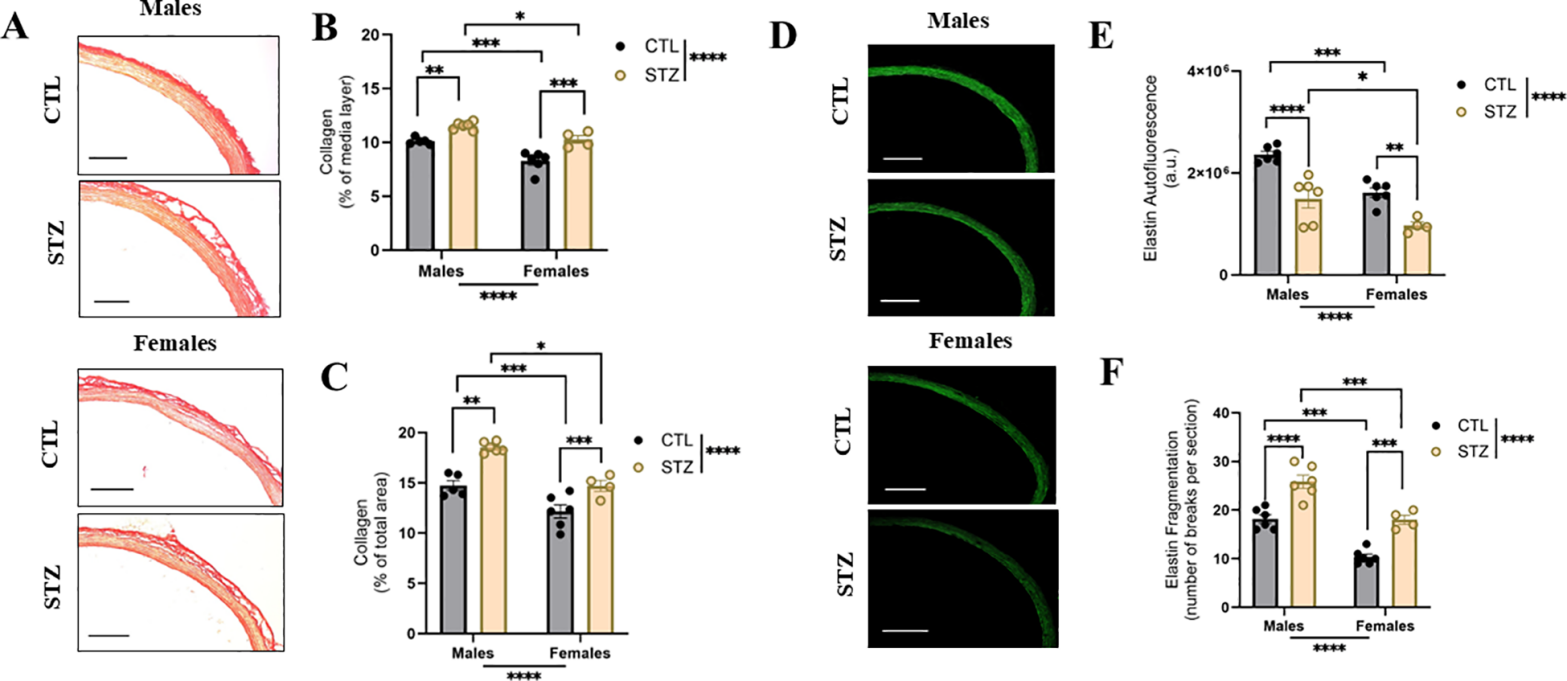
Sex dependent extracellular matrix alterations in the diabetic male and female aorta. **(A)** Representative images of collagen deposition (stained in red), **(B)** collagen (% of positive area within the media layer), **(C)** collagen (% of positive area in total vessel), **(D)** representative images of elastin autofluorescence, **(E)** elastin fluorescence intensity, arbitrary units (a.u.), and **(F)** fragmentation of elastin fiber (number of breaks per section) in aortas of control (CTL) and streptozotocin (STZ)-induced diabetic male and female rats. Images were adjusted for brightness and contrast using the same parameters. Scale bar = 100 μm. Data are shown as mean ± SEM; *p<0.05, **p<0.01, ***p<0.001, and ****p<0.0001 using a two-way ANOVA with Sidak’s *pos -hoc* test, *n* = 4–6 per group.

As key structural determinants of the vascular wall, collagen and elastin levels were assessed in the aortas of control and diabetic animals. In control animals, collagen content was higher in males than in females in both the media layer and across the entire vessel (**Figures 2A, C**). Diabetes significantly increased collagen deposition (% positive area stained in red) in both sexes (**Figures 2A, C**), and this difference relative to sex matched controls (i.e., delta change in collagen, expressed in % point difference) was greater in diabetic males compared to diabetic females (Δ males: 3.79 ± 0.21 *vs* Δ females: 2.54 ± 0.54, n=4-6, p=0.0397).

Elastin degradation was observed in diabetic animals, as indicated by decreased autofluorescence of elastic fibers (**Figures 2D, F**). In addition, diabetic animals showed increased elastin fragmentation, and this difference in alteration (i.e., delta change vs respective controls) was not modulated by sex, (Δ males: 7.67 ± 1.35 *vs* Δ females: 7.67 ± 0.91, n=4-6, p>0.9999) (**Figures 2D, F**). Control animals displayed sex dependent differences in elastin autofluorescence and fragmentation.

### 3.3 Biomechanical assessment of aortic stiffness in T1D males and females

**Figure 3:**
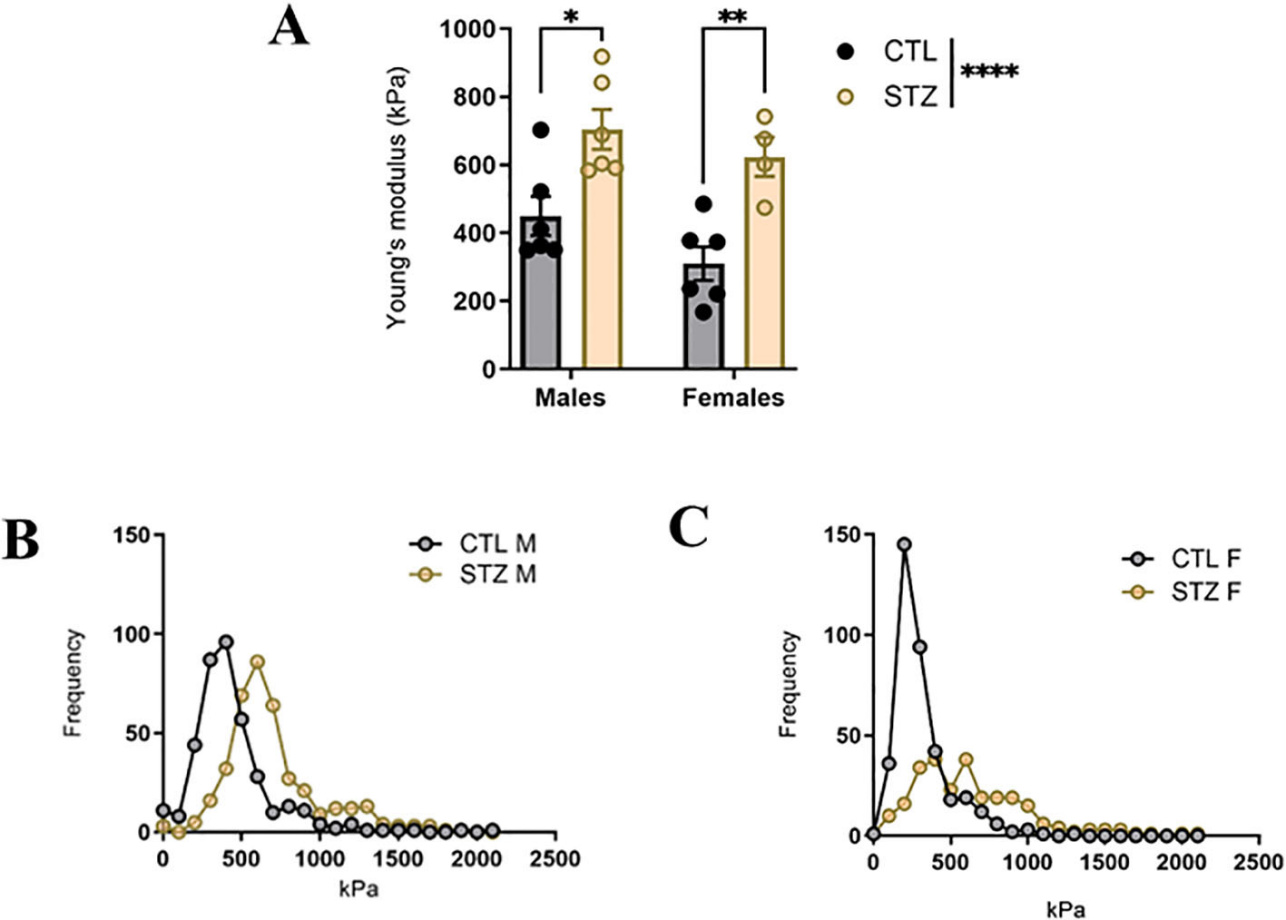
AFM, Atomic force microscopy -based nanoscale assessment of aortic stiffness in control (CTL) and STZ, streptozotocin -induced diabetic male and female rats. **(A)** Young’s Modulus (as measured by AFM) and **(B)** frequency distribution of Young’s modulus in aortas of CTL and STZ male and female rats. Data are shown as mean ± SEM; *p<0.05 and **p<0.01, using a two-way ANOVA with Sidak’s *post hoc* test, *n* = 4–6 per group.

AFM nanoindentation was used to assess intrinsic VSMC stiffness in the aortic tunica media, reflecting biomechanical properties. Young’s modulus was significantly increased in diabetic animals when compared to their respective controls in both males and females. No significant sex differences were observed under control or diabetic conditions (**Figure 3A**). Frequency distribution of Young’s modulus further demonstrated a rightward shift in the stiffness distribution in diabetic animals compared to controls (**Figures 3B, C**).

## 4 Discussion

In this study, we investigated the impact of T1D on arterial stiffness across multiple scales- macro (as measured by PWV), micro- (ECM composition), and nano- (VSMC stiffness using AFM), and assessed whether sex as a biological variable affects these changes. Given evidence showing sex dependent effects in CVD, and the paucity of information about sex differences in the vascular biomechanics, addressing this gap is critical (Mosca et al., 2011; de Oliveira et al., 2022). The STZ-induced model of T1D was used in our study, as it is translational by mimicking human diabetic vasculopathy and is well-established for studying diabetes-associated structural and functional changes in the aorta (Ghasemi and Jeddi, 2023; Rastogi et al., 2025a). Our findings demonstrate that sex differences in arterial stiffness in diabetic animals are evident *in vivo* (at the macro-level), accompanied by ECM remodeling at the micro-level, but no sex-dependent differences in nano-level VSMC stiffness were observed in the aorta of the T1D rat model. These results suggest that sex-dependent differences in macro-level stiffness cannot be explained by intrinsic VSMC stiffness in the aorta but are more likely attributed to sex-specific alterations in the primary components of ECM, such as collagen deposition.

Sex-specific patterns in arterial stiffness and ECM composition have been previously documented under physiological conditions. For instance, some studies have shown higher PWV in males than females (Tomiyama et al., 2003; Chen et al., 2024; Saz-Lara et al., 2025), while others have reported no significant sex-based differences (Díaz et al., 2014), highlighting the complexity of the influence of biological sex in arterial biomechanics. Similarly, in healthy rats, aortic VSMC stiffness (measured via AFM) remains consistent across sexes, despite differences in collagen content, suggesting that cellular stiffness may be independent of a shift in ECM composition (de Oliveira et al., 2022). Further, age- and sex-dependent variations in collagen and elastin content have been reported in healthy mice, suggesting that these differences in ECM components may affect the progression of stiffness over time (Choi et al., 2025). Our study extends these observations to a T1D model, investigating whether diabetes shifts or exacerbates pre-existing sex-dependent differences present under physiological conditions.

At the macro-level, PWV is widely recognized as the gold-standard measure of systemic arterial stiffness, associated with hemodynamic load and pressure conditions, and predictive of early vascular changes (Sugawara et al., 2019; Pilz et al., 2024). Consistent with prior reports showing increased PWV in T1D males (George et al., 2015), we observed elevated PWV in diabetic animals of both sexes compared with controls. Importantly, the increase in PWV induced by diabetes was greater in males than in females, indicating more pronounced central arterial stiffness in males, whereas control animals showed no difference between both sexes. These results suggest that diabetes unmask sex-dependent disparities in stiffness that are not evident under physiological conditions, consistent with a previous study (de Oliveira et al., 2022). In addition, diabetic animals exhibited a smaller aortic diameter relative to controls, with no sex differences in either controls or diabetic animals. Together, these findings indicate that diabetic males exhibit a more pronounced effect of T1D, consistent with clinical observations showing higher PWV in T1D men than in women (Love et al., 2022).

At the micro-level, ECM remodeling was assessed, as the balance between collagen and elastin is a major determinant of arterial mechanical behavior (Wang et al., 2021). Diabetes is known to disrupt ECM composition through mechanisms such as glycation, contributing to arterial stiffening (Searls et al., 2012; Stephen et al., 2014). While diabetes-induced ECM remodeling has been well documented, sex-specific differences in T1D vasculature and their implications for VSMC stiffness remain poorly understood. In our study, diabetes elevated collagen deposition, with diabetic males showing greater accumulation than diabetic females, whereas females exhibited lower collagen levels under both control and diabetic conditions. Elastin loss and fragmentation were also observed in diabetic animals. These results highlight sex-dependent ECM alterations under diabetic conditions, which might contribute to the lower stiffness observed in females *in vivo* using PWV. Notably, under physiological conditions, PWV was comparable between the sexes despite changes in ECM, which may be due to the fact that PWV depends on multiple factors, including vessel radius, thickness, blood density, and endothelial cell function (Yuen et al., 2026).

Following the identification of sex differences at the macro-level and within ECM remodeling, we next assessed whether intrinsic cellular stiffness differed between sexes using AFM-based nanoindentation, which provides high-resolution biomechanical characterization of biological samples (Radmacher, 1997; Bae et al., 2016). Previous AFM-based studies conducted in many cell types, including endothelial and smooth muscle cells under diabetic conditions, have reported increased cellular stiffness, associated with alterations in cytoskeletal and mechano-transduction pathways (Akhtar et al., 2014; Benech et al., 2014; Targosz-Korecka et al., 2017; Zhu et al., 2018; McCallinhart et al., 2020; Wang et al., 2024). Importantly, despite a growing number of AFM-based studies in diabetes, sex as a biological variable remains underexplored, representing a critical gap which was addressed in our study. Our data demonstrate increased Young’s modulus in the aortic media layer of both diabetic males and females compared with controls, confirming diabetes-induced VSMCs stiffening and suggesting a shift in stiffness from physiology to pathology. However, unlike macro- and micro-level findings, no sex differences were observed at the nano-level in the diabetic animals. Prior studies in rats under physiological conditions reported no sex differences at the nano-level despite differences in collagen; our findings extend this to the T1D condition, and our data suggest the absence of sex differences at the nanoscale in our study is a biological effect. To the best of our knowledge, this is the first study to examine sex-based disparities in VSMC stiffness from macro to nano level in the aorta of the T1D model. These findings suggest that intrinsic VSMC stiffness alone is insufficient to account for sex-dependent differences in arterial stiffness at this stage of disease, underscoring the importance of ECM remodeling. Nevertheless, this does not exclude the possibility that VSMC stiffness could be an important marker of sex-specific CVD risk at a later disease stage.

The lower stiffness observed *in vivo* in diabetic females as compared to males aligns with reduced ECM remodeling observed. In this case, the fact that females are less affected may partly be attributed to the influence of sex hormones. For instance, primary female hormones such as 17β-estradiol and progesterone have been shown to reduce collagen deposition and increase elastin content in human aortic smooth muscle cells, which might contribute to ECM preservation in diabetic females (Natoli et al., 2005). However, we acknowledge the limitations of our study, such as the fact that estrous cycle phase or hormone levels were not evaluated, which could provide additional mechanistic insights into sex specific remodeling.

Clinical studies using brachial-ankle PWV show that men have higher stiffness than females with normal glucose regulation; however, in diabetes, this difference is diminished, particularly in older adults (Zhang et al., 2023). Whereas our rat model shows higher aortic PWV in diabetic males early in T1D, highlighting that sex differences in arterial stiffness may vary by PWV measurement site, disease stage, and age. Interestingly, existing literature reports that women with T1D have a 40% higher risk of developing all-cause mortality and vascular complications compared with men (Huxley et al., 2015; Simmons, 2015; Mesa et al., 2025). However, macrovascular function is not always worse in women with T1D, when considering also age as a factor, suggesting that microvascular dysfunction in young diabetic women could be the driving factor behind increased CVD risk (Love et al., 2022).

Altogether, our findings suggest that T1D affects the aortic structure at multiple scales, with sex-based differences observed at the macro- and micro- but not at the nano-level. Diabetic males showed greater progression and more pronounced structural alterations than diabetic females. Thus, our data suggest that T1D exerts differential effects on arterial stiffness as evidenced by elevated PWV in a sex-dependent manner, with changes in ECM composition as a possible contributing mechanism. However, no detectable sex differences at the cellular level in diabetes may indicate early arterial remodeling as depicted in our study, with sex-specific cellular alterations emerging later. Further studies are required to determine whether nano-level biomechanical changes develop at later stages of disease progression, following macro- and micro-level alterations.

## Data Availability

The original contributions presented in the study are included in the article/supplementary material. Further inquiries can be directed to the corresponding author.
